# Will I Fit in and Do Well? The Importance of Social Belongingness and Self-Efficacy for Explaining Gender Differences in Interest in STEM and HEED Majors

**DOI:** 10.1007/s11199-016-0694-y

**Published:** 2016-10-29

**Authors:** Una Tellhed, Martin Bäckström, Fredrik Björklund

**Affiliations:** Department of Psychology, Lund University, Box 213, SE-221 00 Lund, Sweden

**Keywords:** Gender, Interest, Belongingness, Self-efficacy, STEM, HEED

## Abstract

Throughout the world, the labor market is clearly gender segregated. More research is needed to explain women’s lower interest in STEM (Science, Technology, Engineering and Mathematics) majors and particularly to explain men’s lower interest in HEED (Health care, Elementary Education, and the Domestic spheres) majors. We tested self-efficacy (competence beliefs) and social belongingness expectations (fitting in socially) as mediators of gender differences in interest in STEM and HEED majors in a representative sample of 1327 Swedish high school students. Gender differences in interest in STEM majors strongly related to women’s lower self-efficacy for STEM careers and, to a lesser degree, to women’s lower social belongingness expectations with students in STEM majors. Social belongingness expectations also partly explained men’s lower interest in HEED majors, but self-efficacy was not an important mediator of gender differences in interest in HEED. These results imply that interventions designed to lessen gender segregation in the labor market need to focus more on the social belongingness of students in the gender minority. Further, to specifically increase women’s interest in STEM majors, we need to counteract gender stereotypical competence beliefs and assure women that they have what it takes to handle STEM careers.

Throughout the world, the labor market is clearly gender-segregated (Anker 1998; Bureau of Labor Statistics 2015; European Commission 2014). The segregation is apparent both horizontally—simplified as women working with “people” and men with “things” (Su et al. 2009)—and vertically, with men dominating superior positions across sectors (Blackburn et al. 2014). This is true even in relatively gender-equal countries, including Sweden (European Commission 2009, 2014; World Economic Forum 2014), where the present study was performed. The aim of the current study is to explore explanations of horizontal gender segregation in the labor market, which we do by testing for mediation of gender differences in high-school students’ educational interests.

Gender segregation in the labor market has been identified as creating recruitment problems for employers, perpetuating the undervaluation of female-dominated work and limiting individuals’ career opportunities (European Commission 2014). It is therefore important to clarify why so few men are attracted to “people” careers (e.g., health care and education) and so few women are attracted to “things” careers (e.g., science and engineering). In other words, why do the career interests of young men and women differ?

## HEED and STEM

Whereas the reasons for women’s lack of interest in the male-dominated STEM (Science, Technology, Engineering and Mathematics; Koch et al. 2014) sector are relatively well understood, this is not the case for men’s lack of interest in female-dominated careers (Croft et al. 2015; Watt 2010), which have recently been termed HEED (Health care, Elementary Education and the Domestic sphere; Croft et al. 2015; Watt 2008, 2010). An important aim of the current study is therefore to test mediation of gender differences in interest in the HEED sector and investigate if it differs from mediation of gender differences in interest in the STEM sector. There is reason to belive that mediation of these gender differences will partly differ because structural gender inequalities in society (Ridgeway 2001) and their associated gender stereotypes (Eagly 1987; Fiske et al. 2002) impact men and women (Rudman and Glick 2008a), and may cause their career concerns to differ, which will be explained in more detail in the following.

We base our career interest hypotheses on the well-established social cognitive career theory (SCCT; Lent et al. 1994), which states that career interests are influenced by beliefs of what we *can do* (self-efficacy) and what we *will get* (outcome expectations) in relation to career opportunities (Lent et al. 1994; Lent and Brown 2006). We will test for gender differences in self-efficacy and one novel outcome expectation, social belongingness, and investigate their importance for explaining gender differences in high school students’ interest in STEM and HEED majors.

## Self-Efficacy

*Self-efficacy* is defined as the belief that one has the capability to succeed in a domain (Bandura 1977). The lion’s share of research on career interest has focused on self-efficacy, and it is the most central concept of SCCT. According to Bandura (1997), we tend to approach domains in which we feel competent and avoid those in which we do not. Women tend to have lower self-efficacy than men do for STEM occupations like engineering (see Hackett 1995 for a review) and for STEM-relevant abilities, such as math (Bench et al. 2015; Betz and Hackett 1983; Else-Quest et al. 2010; the Organisation for Economic Co-operation and Development (OECD) 2015). It thus follows that women are less attracted to STEM careers than are men, and self-efficacy has been shown to be an important mediator of gender differences in interest in STEM careers (Eccles 1987, 1994; Eccles et al. 1983; Hackett 1995; Lent et al. 1994, 2008b; Rottinghaus et al. 2003).

What about men’s self-efficacy for HEED careers? This is less well researched. Previous studies have shown that women believe they will do well in female-dominated HEED careers (e.g., teaching), but they have doubts concerning their abilities for male-dominated STEM careers (e.g., engineering). Men, on the other hand, tend to believe they can handle male- and female-dominated careers equally well (Betz and Hackett 1981; Bridges 1988; Matsui et al. 1989). One reason for men’s lesser self-efficacy concerns may be found in structural gender inequality in society which affords lower status to women. According to social role theory (Eagly 1987) and stereotype content theory (Fiske et al. 2002), this structure shapes the content of gender stereotypes, which associate men with competence and women with warmth (Fiske et al. 2002).

The difference in men’s and women’s association with competence is likely to have implications for how men and women develop their self-efficacy. According to SCCT, people’s self-efficacy beliefs are shaped by their learning experiences within a domain (Lent et al. 1994), which consist of performance accomplishments, vicarious learning, verbal persuasion, and physiological arousal (Bandura 1997). Men and women have been shown to have fewer learning experiences in domains that are dominated by the gender outgroup (Williams and Subich 2006). Also, when men and women have similar learning experiences, there is reason to believe that they may affect their self-efficacy asymmetrically, which causes women to have lower self-efficacy than men do. For example, Bandura (1997) states that perceptions of previous success and failure in a domain (performance accomplishments) strongly affect our self-efficacy in that domain. However, even though meta-analyses tend to show gender similarity in most abilities (e.g., Hyde 2014), the stereotypical association of competence with men puts women at risk of undervaluing their performance and men of overvaluing it (Bench et al. 2015; Watt 2010). Thus, when men and women perform similarly, women’s resulting self-efficacy may still be lower than men’s. There is a lack of studies investigating the importance of gender differences in self-efficacy for explaining gender differences in interest in the female-dominated HEED sector, which we aim to do in the current study. Because men seldom have self-efficacy concerns for female-dominated occupations, we expect smaller gender differences in self-efficacy for HEED majors, which means that self-efficacy should be a less important mediator of gender differences in interest in HEED as compared to gender differences in interest in STEM. To understand men’s low interest in HEED, we next turn to the second proposed factor behind career interest in SCCT: outcome expectations.

## Outcome Expectations

*Outcome expectations* represent our beliefs about the consequences that different career choices have, such as salary expectations or consequences for work-family balance or self-evaluation (Lent et al. 1994; Lent and Brown 2006). Both self-efficacy and outcome expectations matter for career interest. For instance, although many women have low self-efficacy beliefs for STEM careers, this does not imply that they necessarily perceive these careers as low in value (Schmader et al. 2001). In fact, STEM careers are relatively high in status (Cohen and Huffman 2003; Croft et al. 2015; Svensson and Ulfsdotter Eriksson 2009).

There is not much empirical work on outcome expectations in relation to career interest and the results are mixed: Some studies have found support for SCCT’s prediction that they relate positively (Lent et al. 2010), but others have not (Lent et al. 2008a). Outcome expectations have been proposed to matter also for gender differences in career interest (Croft et al. 2015). One aspect of structural gender inequality is that female-dominated work is lower in status than male-dominated work is (Cohen and Huffman 2003; Croft et al. 2015; Svensson and Ulfsdotter Eriksson 2009). According to social role theory, this structural status difference has resulted in the stereotype of men as more status-driven (agentic) than women are so that men are a better fit for high-status careers (Cejka and Eagly 1999; Eagly and Karau 2002; Heilman and Kram 1983). Correspondingly, female-dominated careers are perceived as affording communal goal fulfillment (e.g., helping, Diekman et al. 2010, 2011) which is stereotypically associated with women (Abele and Wojciszke 2007; Bakan 1966).

Interestingly, Diekman et al. (2010, 2011) have found that women have higher communal career goals (e.g., wanting to help others, work with people) than men do and that this partially mediates gender differences in interest in STEM careers because they appear to afford little communal goal fulfillment (an outcome expectation). There is however no support for agency goals as a mediator of the gender difference in interest in the HEED sector (Diekman et al. 2010, 2011), which perhaps could be expected because HEED careers afford low goal fulfillment of agency and men are stereotypically portrayed as more agency-oriented (e.g. desiring status, power and recognition,) than women are (Diekman et al. 2010, 2011). However, for a factor (agency) to mediate a gender difference in interest, there must be a gender difference in that factor, and perhaps surprisingly, there is no empirical support for a gender difference in agency (Diekman et al. 2010, 2011; Twenge 1997, 2001). This calls for a search for gender-relevant outcome expectations that may explain gender differences in interest also in HEED majors. In the present study we will investigate the possibility that gender differences in social belongingness expectations in STEM and HEED majors may partly explain gender differences in interest in them.

## Social Belongingness

*Social belongingness* is defined as the perception of social connectedness in groups, that is, of fitting in socially with others (Baumeister and Leary 1995; Walton and Cohen 2007). Social belongingness has been recognized as a fundamental human need, underlying much of our psychological functioning (Baumeister and Leary 1995). According to social identity theory (Tajfel and Turner 1979), group living has been essential for survival in human history and has accordingly shaped much of our psychology. We are attracted to people we perceive as similar to us—our ingroup (Montoya and Horton 2012), expect to belong better in ingroup-dominated contexts (Easterbrook and Vignoles 2013), and favor our ingroup over outgroups (Brewer 1999). Social belongingness concerns are especially heightened during adolescent years, which coincide with the making of important career choices (Somerville 2013).

Because social belongingness is so important, especially for adolescents, it appears reasonable that high-school students who ponder alternative career paths may reflect upon expected outcomes in terms of social belongingness, in addition to other outcome expectations they find important. For instance, when considering what major to apply to, they may be more attracted to, and thus interested in, majors they perceive as dominated by an important ingroup.

People have multiple social identities. What specific ingroup-dominated career context one is attracted to is likely to vary depending upon what ingroup one identifies with when the career choice is to be made. However, there is reason to believe that our gender ingroup has a special standing among our social identities, especially for younger individuals. Research has shown that gender tends to be the first social category children learn (see Mehta and Strough 2009; Rudman and Glick 2008b for reviews) and that once they obtain their gender identity, they typically develop strong gender ingroup favoritism (Rudman and Glick 2008b). In sum, young children generally become *homosocial*, preferring (non-sexual) relationships with people of the same gender (Lipman-Blumen 1976; Rudman and Glick 2008b), and this tendency often grows stronger throughout childhood (Rudman and Glick 2008b). Gender segregation in childhood is generally so strong that it has been suggested that boys and girls grow up in different cultures (Maccoby 1998).

According to SCCT, learning experiences influence outcome expectations (Lent et al. 1994; Williams and Subich 2006), and because children lead gender-segregated lives, there is reason to think that they will expect better social belongingess in gender ingroup-dominated careers, which thereby attracts their interest. To our knowledge, social belongingness expectations have not previously been tested as mediators of gender differences in career interest, but a few studies have shown that it relates to motivation and performance in school (Walton and Cohen 2007; Walton et al. 2015). Also, Cheryan and Plaut (2010) have found that gender differences in psychology students’ perceived *similarity* to students majoring in English versus Computer Science was a mediator of gender differences in interest in these majors. The authors discussed that perhaps students are attracted to majors where people appear similar to themselves because they expect to belong better with similar people. Further, a couple of interesting studies have shown that women’s sense of ambient belonging in stereotypical versus non-stereotypical STEM classroom environments affects their interest in STEM (Cheryan et al. 2009, 2011).

In light of this reasoning, we hypothesize that high-school students expect to belong better socially in majors that are dominated by same gender peers. In other words, we predict that young men will expect to fit in better socially in male-dominated STEM majors than in female-dominated HEED majors and vice versa for women. Furthermore, gender difference in expected social belongingness in STEM and HEED majors should partially mediate gender difference in interest in them.

## The Present Study

To summarize, in the present study we will test for gender differences in high-school students’ self-efficacy and social belongingess expectations in relation to STEM and HEED majors and then test if this mediates gender differences in interest in them. We will also compare the importance of self-efficacy versus social belongingness expectations as potential mediators of career interest in STEM versus HEED. This comparison is important because policymakers need to know what to prioritize when designing efficient interventions for reducing gender segregation in the labor market. We propose four main hypotheses: (a) There are gender differences in interest in STEM majors and HEED majors (Hypothesis 1), (b) There are gender differences in self-efficacy as well as social belongingness expectations in relation to STEM majors and HEED majors (Hypothesis 2), (c) Gender differences in self-efficacy and social belongingness expectations mediate gender differences in interest (Hypothesis 3), and (d) Self-efficacy and social belongingness differ in the extent to which they mediate a gender difference in interest in STEM majors and HEED majors (Hypothesis 4). Finally, although we expected STEM majors to be regarded as higher in status than HEED majors were among high-school students (Cohen and Huffman 2003; Croft et al. 2015; Svensson and Ulfsdotter Eriksson 2009), and status is likely to relate to interest, we did not expect gender differences in the status ratings. Therefore status is not a proposed mediator of gender differences in interest.

## Method

### Participants and Procedure

Fully 1327 Swedish senior high-school students in university-preparatory programs participated in the present study (650 males and 677 females, *M*_age_ = 18.89, *SD* = .05). To obtain a representative sample of the Swedish population, we randomly selected 12 municipalities from a national database representing three “big cities” (*n* = 505), eight “cities” (*n* = 776), and one “sparsely populated area” (*n* = 46) and contacted high schools there (Sveriges Kommuner och Landsting (SKL) 2010). The goal sample sizes were set in proportion to the number of 17-year-olds residing in the municipality 1 year prior to our data collection according to national statistics (Statistics Sweden 2015). The data were collected in the schools where the personnel had assembled the students for our visit. We informed the students that the study investigated psychological explanations of young people’s career interests and that the data would be analyzed only on a group level, comparing for instance different age groups, gender groups, and residential areas. We did not mention that gender differences were the specific main interest of the study to avoid priming them with gender. Participants gave informed consent and filled out a questionnaire using pen and paper. They were given one ice cream gift card and one movie ticket for participating. The study has been approved by the Regional Ethical Review board in Lund, Sweden.

Because our study investigates the relationship between the participant’s legally assigned gender (male or female) and their perceptions of university majors, we use participant gender as a dichotomous independent variable in the current study, although gender identification is not dichotomous in the population (Dreger 2008). Using data from Sweden’s Higher Education Authority (Universitetskanslerämbetet (UKÄ) 2013), we created two categories of university majors from the largest, gender-skewed university majors in Sweden: (a) STEM (Engineering, 73 % men; Computer Programming, 76 % men) and (b) HEED (Nursing, 14 % men; Preschool Teacher, 8 % men; Primary school teacher, 24 % men).

### Measures

The measures presented in the current article represent a subset of a larger dataset collected in a research project financed by the Swedish Research Council for Health, Working Life and Welfare (grant # 2012-0435). For the larger project, data have also been collected for a parallel sample of younger participants (15-year-olds); the project measures many aspects of career fit that may explain educational gender segregation. For a full list of measures in the larger project, please see the public grant application and ethics application (# 2013-266). For the present analyses, participants rated interest, self-efficacy, social belongingness, and status for each of the selected university majors. The ratings for each variable were summed according to the major categories STEM (engineering, computer programming) and HEED (nursing, preschool teacher, primary school teacher) to form an indication of a common factor (i.e., an indication of interest in STEM and HEED majors). Because individuals’ ratings were expected to vary across the five majors, we did not calculate alphas for our STEM and HEED variables; rather, these variables represent a general assessment of each individual’s interest in, projected self-efficacy in, perceived social belongingness in, and judged status of our STEM and HEED categories.

For *interest,* participants rated how interested they were in studying the five different majors on a scale from 1 (*not at all*) to 7 (*extremely*), similar to interest measures in Lent et al. (2001) and Diekman et al. (2010, 2011). For *self-efficacy* participants rated how certain they were that they could successfully complete the work tasks demanded in the five different occupations associated with the majors (e.g., in engineering, nursing) on a scale from 1 (*not at all certain*) to 7 (*completely certain*). This operationalization is congruent with the recommendations for measuring self-efficacy within social cognitive career theory (Lent and Brown 2006) and expectancy-value theory (Betz and Hackett 1981; Maurer and Pierce 1998). For *social belongingness*, participants rated their belief in how well they would fit in socially with their classmates if they were students in the majors on a scale from 1 (*not fit in at all*) to 7 (*fit in extremely well*). This item represents one item in the SCCT Support and Barrier scale used in Lent et al. (2001), and it is congruent with other belongingness measures in the literature (e.g., Cheryan et al. 2009; Good et al. 2012). Lastly, participants rated the *status* of the majors on a scale from 1 (*lowest possible status*) to 5 (*highest possible status*).

## Results

### Gender Differences in Interests

We first tested Hypothesis 1, which predicted that men are more interested than women are in STEM majors and that women are more interested than men are in HEED majors, using mixed ANOVA with STEM and HEED ratings as a within-subject factor and gender as between group factor. As predicted, the interaction between major category (STEM, HEED) and participants’ gender on interest was strong, *F*(1, 1325) = 404.56, *p* < .001, η_p_^2^ = .234. Male participants were much more interested in the STEM majors than the female participants were, and female participants were more interested in HEED majors than the male participants were, although this latter difference was smaller (see Table 1). The effect size was large for STEM majors and medium for HEED majors. Thus Hypothesis 1 was supported.

**Table 1:** Descriptive statistics of interest for STEM and HEED majors

	Men		Women				
Variables	*M*	*SD*	*M*	*SD*	*t(*1325)	Cohen’s *d*	*M*_Diff_
Interest							
STEM	3.74	1.74	2.19	1.35	18.14	1.00	1.55^***^
HEED	1.66	.94	2.18	1.22	8.71	−.48	−.52^***^
Belongingness							
STEM	4.53	1.51	3.36	1.53	14.01	.77	1.17^***^
HEED	3.60	1.44	4.36	1.46	9.54	−.52	−.76^***^
Self-efficacy							
STEM	4.37	1.57	3.16	1.53	14.14	.78	1.21^***^
HEED	4.34	1.50	4.74	1.51	4.85	−.38	−.27^***^

*M*_Diff_ = *M*_men_ - *M*_women_

^***^ *p* < .001

### Self-Efficacy and Social Belongingness

Social belongingness and self-efficacy were hypothesized as potential mediators of gender mean differences in interest in STEM and HEED majors. We first tested for gender differences for the proposed mediators (Hypothesis 2). There were clear gender differences (see Table 1) in social belongingness and self-efficacy in relations to the STEM majors (medium effect sizes) and HEED majors (small effect size for self-efficacy and medium for belongingness).

Table 2 displays the correlations of the proposed mediators with the dependent variable (interest). Both social belongingness and self-efficacy were related to interest in STEM and HEED majors. The table shows that self-efficacy was more strongly related to interest in STEM as compared to HEED majors. Likewise, social belongingness was also more strongly related to interest in STEM majors as compared to HEED majors.

**Table 2:** Correlation among study variables for STEM and HEED majors

	Belonging		Self-Efficacy		Interest	
	STEM	HEED	STEM	HEED	STEM	HEED
Belonging						
STEM	–					
HEED	.09**					
Self-efficacy						
STEM	.69***	−.10**	–			
HEED	.03	.43***	.18***	–		
Interest						
STEM	.63***	−.14**	.69***	−.03	–	
HEED	−.15***	.45***	−.17***	.30***	−.08**	–

* *p* < .05. ** *p* < .01. *** *p* < .001

### Mediation

To test for mediation of gender differences in interest, we estimated the indirect and direct effects with the MPlus program (version 7.11) making it possible to simultaneously estimate more than one mediator (self-efficacy and social belongingness). Because men were coded as 1 and women as 2, positive indirect effects suggest that women had higher ratings. There were significant indirect effects for belongingness for both interest in STEM, with a point estimate of −.311 (99 % CI [−.440. −.211]), and HEED majors, with a point estimate of .204 (99 % CI [.137, .289]). This suggests that belongingness is a possible mediator of gender differences in interest for both HEED and STEM majors. Self-efficacy had a rather strong indirect effect to interest for STEM majors, with a point estimate of −.561 (99 % CI [−.724, −.433]), but the indirect effect for HEED majors was weak, with a point estimate of .037 (99 % CI[.015, .068]). This suggests that self-efficacy is a strong possible mediator of gender differences in interest in STEM majors, but not so for HEED majors.

Because the main aim of the study was comparing the strength of mediators, we also tested the size of the indirect effects using Model constraints; that is, we tested directly if two indirect effects were significantly different by calculating the indirect effects and then testing the difference between them. These tests showed that self-efficacy was the strongest mediator of gender differences in interest in STEM majors, stronger than belongingness (∆*b* = .250, 99 % CI [.053, .438]). The mediation tests for gender differences in interest in HEED majors revealed that belongingness had the strongest indirect effect. The indirect effect of belongingness was larger than the effect from self-efficacy (∆*b* = .167, 99 % CI [.101, .253]).

The total mediation of STEM and HEED was only partial, with participants’ gender still having a direct effect on interest to STEM majors, with a point estimate of −.677 (99 % CI [.859, −.485]), and HEED majors, with a point estimate of .279 (99 % CI[.139, .439]). Thus, our mediation hypotheses (Hypotheses 3 and 4) were supported: Self-efficacy partially mediated the gender difference in interest for STEM majors and the effect was much larger than for HEED majors, whereas social belongingness partially mediated the gender difference in interest for both HEED and STEM majors.

### Other STEM and HEED Gender Differences

To facilitate understanding of the mediation results, we now turn to some more descriptive results. The main effects for interests differed between programs. As seen in Table 1 testing the within-subject factor, we found that interest was generally higher for STEM majors as compared to HEED majors, ∆*M* = .51, *F*(1, 1325) = 412.18, *p* < .001, η_p_^2^ = .237. Also, using simple effects to test the interaction, the men’s interest was found to differ much more between STEM and HEED, ∆*M* = 2.08, *F*(1, 1325) = 800.43, *p* < .001, η_p_^2^ = .382, as compared to the women’s interest for STEM and HEED, ∆*M* = .01, *F*(1, 1325) = .02, *p* > .001, η_p_^2^ < .001. This indicates that men had more gender stereotypical educational interests than women did.

Further, women had much stronger self-efficacy in relation to HEED majors as compared to STEM majors, ∆*M* = 1.58, *F*(1, 1325) = 478.90, *p* < .001, η_p_^2^ = .265; whereas the difference for men was close to zero, ∆*M* = −.03, *F*(1, 1325) = .153, *p* > .001, η_p_^2^ < .001. Thus only women had gender-stereotypical self-efficacy, whereas men believed they could successfully complete the work tasks demanded in HEED, as well as those in STEM, occupations. Thus, when self-efficacy mediates mean differences in interest to STEM, this mediation can be attributed to women’s more varied self-efficacy.

Both women and men anticipated much stronger social belongingness in gender ingroup dominated majors: Female participants expected greater belongingness in HEED majors as compared to STEM majors, ∆*M* = 1.00, *F*(1,1325) = 191.92, *p* < .001, η_p_^2^ = .127, and male participants expected greater belongingness in STEM majors as compared to HEED majors, ∆*M* = .93, *F*(1,1325) = 157.38, *p* < .001, η_p_^2^ = .106. Accordingly, mediation can be attributed to variation in belongingness for both men and women.

Lastly, we tested for differences in status between STEM and HEED majors. Participants rated the HEED majors as much lower in status, (*M*_total_ = 2.21, *SD* = .74) as compared to STEM majors (*M*_total_ = 3.66, *SD* = .73). The difference was very large, *t*(1325) = 34.00, *p* < .001, *d* = 1.34. Status was related to interest in STEM majors, *r*(1326) = .231, *p* < .001, and HEED majors, *r*(1326) = .198, *p* < .001, but status did not mediate differences in interest between young men and women. (As expected, there were no significant gender differences in ratings of status. The β weights of gender differences in interest were not affected by adding status to the model).

## Discussion

The aim of the current study was to test if self-efficacy beliefs and social belongingness expectations mediate gender differences in high-school students’ interest in STEM majors (e.g., engineering) and HEED majors (e.g., nursing). It has not been previously elucidated why men are less interested in HEED careers than women are, but our results suggest that part of the explanation lies in adolescents’ homosocial belongingness expectations. Both male and female high-school students expected to belong much better in majors that were dominated by same-gender peers; women in HEED and men in STEM. The gender difference in social belongingness expectations partially mediated the gender difference in interest in both kinds of major. Thus, part of the reason why women are more interested in HEED majors than men are, and men more interested in STEM majors than women are, is related to adolescents’ social belongingness concerns.

As a further contribution to the literature we showed that self-efficacy is a more important mediator of gender differences in interest in STEM majors as compared to HEED majors. Replicating previous studies (Betz and Hackett 1981; Bridges 1988; Matsui et al. 1989), female participants had gender-stereotypical self-efficacy beliefs, where they were much more confident in their ability to handle HEED careers, as compared to STEM careers, whereas men were equally confident in their ability to handle both types of careers. Our results suggest that although interventions designed to increase women’s interest in STEM careers should prioritize increasing women’s self-efficacy for STEM, it should be more effective to focus on increasing men’s social belongingness expectations in HEED majors, rather than their self-efficacy, to increase men’s interest in HEED.

Before we discuss these results in more detail, we should point out one unexpected finding in our study: Surprisingly, even though women had higher interest in HEED majors than men did, women actually rated their interest in HEED majors as low as they rated their interest in STEM majors. Perhaps this reflects the low status of HEED majors (Cohen and Huffman 2003; Croft et al. 2015; Svensson and Ulfsdotter Eriksson 2009), which was replicated in the current study. It is possible that the low status of HEED majors makes them unattractive to both men and women. Previous studies have not found gender differences in the desire for career status (Diekman et al. 2010, 2011), and our participant’s status ratings of the majors related to their interest in them. It is conceivable that increasing the status of HEED careers increases both men’s and women’s interest in them.

### Self-Efficacy

Self-efficacy was an important mediator of gender differences in interest in STEM majors, which replicates previous results (Betz and Hackett 1983; Else-Quest et al. 2010; Hackett 1995). It is disturbing that in one of the most gender equal countries in the world (World Economic Forum 2014), where female students have long outperformed male students across school subjects (OECD 2015), young women still have highly gender stereotypical self-efficacy beliefs. How can this be? It appears that women’s competence doubts for male stereotypical domains largely stem from gender stereotypes, where competence is still associated more strongly with men than with women (Fiske et al. 2002). Research shows that when an individual is stereotyped as lower in competence in a domain, stereotype threat may ensue, which gives rise to self-doubts, lower performance expectations, and many other negative effects (Inzlicht and Schmader 2012, for a review). Because self-efficacy is important for career interest, it seems that to increase women’s interest in STEM careers, we need to address gender stereotypes of competence.

In contrast to women, men’s self-efficacy was not dependent on career type. This is interesting because female-dominated careers are stereotypically seen as more fitting for women (Cejka and Eagly 1999; Eagly and Karau 2002; Heilman and Kram 1983). However, men seldom suffer from gender-related stereotype threat (Pillaud et al. 2015), and their self-efficacy may be protected by the strong association between male gender and competence (Fiske et al. 2002). That self-efficacy is less important for explaining men’s lower interest in HEED majors than women’s lower interest in STEM majors is a valuable finding, especially as we need to clarify priorities for interventions aimed at reducing gender segregation in the labor market.

### Social Belongingness Expectations

Social belongingness turned out to be a better mediator of gender differences in interest in HEED majors than self-efficacy was. Social belongingness also mediated gender differences in interest in STEM majors, although self-efficacy was a better mediator of this gender difference. This finding is a new contribution to the research field, and it adds to the understanding of why men and women are attracted to gender-ingroup dominated careers. Both men and women believed they would fit in much better in majors dominated by same gender peers, as compared to majors dominated by the gender outgroup. Social belongingness is considered a fundamental human need (Baumeister and Leary 1995) that especially tends to preoccupy the minds of adolescents (Somerville 2013). This may limit their career consideration to mainly ingroup-dominated careers.

The greater attraction to majors dominated by the gender ingroup may be due to gender ingroup favoritism, or homosociality (e.g., Chatman and O’Reilly 2004; Ibarra 1992; Lipman-Blumen 1976; Rose 1985; Rudman and Goodwin 2004; Rudman and Glick 2008b). Boys and girls grow up gender-segregated, and although the lack of interest in the gender outgroup typically shifts for heterosexual individuals when they enter puberty (Rudman and Glick 2008b), adults too typically prefer to interact with same-gender people (e.g., Chatman and O’Reilly 2004; Ibarra 1992). Gender ingroup favoritism has seldom been discussed as an explanation for horizontal gender segregation, but the current results suggest that it should, particularly if we want people to be more open to gender-atypical career choices.

It is important to note that the process leading up to the belongingness expectations in gender-segregated majors is likely to be more complicated than the simple preference of same-gender peers. Future studies may therefore want to examine how gender differences in social belongingness expectations form in relation to gender skewed majors. For instance, when people consider social belongingness with HEED and STEM students, they are likely to activate a stereotype of what HEED students and STEM students are like, such as what their interests are and what goals and hobbies they have. These stereotypes are likely to overlap with the content of gender stereotypes (Cejka and Eagly 1999; Eagly and Karau 2002; Heilman and Kram 1983), and because most children are socialized to prefer the stereotypes associated with their gender group (goals, interests etc.; Rudman and Glick 2008a), they are likely to perceive greater similarity, and thereby social belongingness, to same-gender students, in addition to the homosocial tendencies they may have.

It may also be relevant to investigate how gender differences in learning experiences (Bandura 1997) influence how the gender-stereotypical social belongingness patterns develop. For instance, how important are past experiences of homosocial and heterosocial interactions for expectations of greater belongingness in STEM versus HEED? Williams and Subich (2006) have shown that learning experiences matter for the general outcome expectation in gender-segregated careers (i.e. the perception that one will get what one wants), but more work is needed.

### Limitations and Future Research Directions

There are several limitations to the present study. The most important methodological limitation is the correlational (cross-sectional) design of the study, which means that the results must be interpreted with caution, because they cannot be inferred to imply causality. We therefore encourage replications with experimental designs that manipulate social belongingness and self-efficacy to determine how it impacts men’s and women’s interest in STEM and HEED majors. Further, the statistical models did not fully mediate the gender differences in educational interest in the study, which implies that more gender-relevant factors contribute to shape gender differences in interests in STEM and HEED majors, in addition to the ones that were tested here. We recommend replications with measures of other outcome expectations, such as expectations of having one’s communal goals met in STEM versus HEED majors (Diekman et al. 2010, 2011). Also, because of time limitations in data collection, we only have single-item measures of self-efficacy and social belongingness (for each educational program), which calls for a more thorough future investigation of these variables with full-scale measures.

Another limitation of our study is that the data were collected in a single country (Sweden), and cross-cultural replications are desirable to distinguish potential universal effects from culture-specific ones. Sweden stands out as low on Hofstede’s cultural value dimension “Masculinity” (Hofstede 2016). This means that Swedes generally value agency goals, such as competition, achievement, and financial rewards, much less than other peoples. When agency goals are valued higher than in Sweden, the differences in interest between low-status HEED and high-status STEM may be even larger. On the other hand, it has surprisingly been shown that in relatively more gender-equal and highly developed countries, like Sweden (United Nations Development Programme 2014; World Economic Forum 2014), gender differences in self-concepts are often larger than elsewhere (see Guimond et al. 2013 for a review). Finally, it is also desirable to replicate our study with participants of different age groups, such as college students. Social belongingness concerns are typically intensified during adolescence (Somerville 2013) and may be less relevant in older samples.

### Practice Implications

One of the reasons for comparing the importance of self-efficacy and social belongingness as mediators of gender differences in educational interest was to provide policymakers with information that can give guidance in the design of efficient interventions for reducing gender segregation in the labor market. Based on our results, we suggest that interventions that specifically focus on increasing girls’ and young women’s interest in STEM majors (e.g., engineering), should prioritize increasing their self-efficacy because this seem to be the most important mediator of gender differences in interest in STEM majors. Competence should be made less masculine-stereotyped to lessen the risk of stereotype threat and self-efficacy doubts in women. Being stereotyped as less competent by society implies that girls and women need more encouragement to develop a secure self-efficacy. For the aim of increasing boys’ and men’s interests in HEED, self-efficacy interventions seem less important because young men seem to believe in their ability to handle the demands of HEED careers.

We further suggest that interventions should focus on increasing young peoples’ expectations of social belongingness in gender-segregated majors because this variable mediated gender differences in interest both in HEED and STEM majors. Academia may, for example, want to focus more on helping gender-outgroup students make friends at university and communicate to potential applicants that students are respected and accepted, regardless of gender identity. It is promising that Walton et al. (2015) have shown that a brief social belongingness intervention made female STEM students make more male friends and receive better grades. The intervention consisted of simply emphasizing the commonness of having social belongingness concerns when entering an engineering class, but that these concerns dissipate with time. Our results point to the importance of these types of interventions.

In the bigger picture, we may want to consider encouraging more cross-gender friendships in children to try and lessen the strong homosociality that is typically evident in childhood (Rudman and Glick 2008b). If gender-integrated play becomes the norm in childhood, perhaps more adolescents will expect to belong well in majors that are dominated by their gender outgroup.

### Conclusion

To reduce horizontal gender segregation in the labor market, we need to make men more interested in working with “people” and women with “things.” The results of the current research indicate that we may need to design partly different interventions to reduce gender skewness in STEM majors versus in HEED majors. To specifically increase women’s interest in male-dominated STEM majors (e.g. engineering), it is crucial to assure women that they have what it takes to handle the demands of a STEM career. This entails continuing the battle against gender stereotypes that associate competence more with men than with women. This is however less important for increasing men’s interest in female-dominated HEED majors (e.g., nursing), where increasing a sense of fitting in appears more pressing. In fact, gender-stereotypical social belongingness expectations are part of the explanation of gender differences in interest in both STEM and in HEED majors. Improving the social integration of gender-outgroup students in higher education should take us closer to the goal of gender integration in the labor market.
